# Tapeworm *Diphyllobothrium dendriticum* (Cestoda)—Neglected or Emerging Human Parasite?

**DOI:** 10.1371/journal.pntd.0002535

**Published:** 2013-12-26

**Authors:** Roman Kuchta, Jan Brabec, Petra Kubáčková, Tomáš Scholz

**Affiliations:** 1 Institute of Parasitology, Biology Centre of the Academy of Sciences of the Czech Republic, České Budějovice, Czech Republic; 2 University Hospital Brno, Brno, Czech Republic; George Washington University, United States of America

## Abstract

**Background:**

A total number of 14 valid species of *Diphyllobothrium* tapeworms have been described in literature to be capable of causing diphyllobothriosis, with *D. latum* being the major causative agent of all human infections. However, recent data indicate that some of these infections, especially when diagnosed solely on the basis of morphology, have been identified with this causative agent incorrectly, confusing other *Diphyllobothrium* species with *D. latum*. Another widely distributed species, *D. dendriticum*, has never been considered as a frequent parasite of man, even though it is found commonly throughout arctic and subarctic regions parasitizing piscivorous birds and mammals. Recent cases of Europeans infected with this cestode called into question the actual geographic distribution of this tapeworm, largely ignored by medical parasitologists.

**Methodology and Results:**

On the basis of revision of more than 900 available references and a description and revision of recent European human cases using morphological and molecular (*cox1*) data supplemented by newly characterized *D. dendriticum* sequences, we updated the current knowledge of the life-cycle, geographic distribution, epidemiological status, and molecular diagnostics of this emerging causal agent of zoonotic disease of man.

**Conclusions:**

The tapeworm *D. dendriticum* represents an example of a previously neglected, probably underdiagnosed parasite of man with a potential to spread globally. Recent cases of diphyllobothriosis caused by *D. dendriticum* in Europe (Netherlands, Switzerland and Czech Republic), where the parasite has not been reported previously, point out that causative agents of diphyllobothriosis and other zoonoses can be imported throughout the world. Molecular tools should be used for specific and reliable parasite diagnostics, and also rare or non-native species should be considered. This will considerably help improve our knowledge of the distribution and epidemiology of these human parasites.

## Introduction

Diphyllobothriosis is a human disease caused by fish tapeworms (or broad tapeworms) of the genus *Diphyllobothrium* Cobbold, 1858 (Cestoda: Diphyllobothriidea). It represents the most important fish-borne zoonosis caused by tapeworms [1]. Most of the cases are asymptomatic, but in about one out of five cases, diarrhoea, abdominal pain, or discomfort occurs [1]. Humans get infected by eating raw, insufficiently cooked, or marinated freshwater and marine fish. Increasing popularity of dishes based on raw fish meat, such as sushi, sashimi, carpaccio, or ceviche, significantly increases the risk of acquiring the parasite, even in the most developed countries. As many as 14 species of *Diphyllobothrium* have been described as capable of causing diphyllobothriosis, with *D. latum* (Linnaeus, 1758) being the dominant species in human infections. Together with *D. nihonkaiense* Yamane, Kamo, Bylund et Wikgren, 1986, *D. latum* is also considered to be the most pathogenic for man [1].

Routine diagnostics of human infections are currently based on morphological observations of relatively small eggs with an operculum and/or segments (proglottides) with median genital pores. Such cases are mostly identified as *D. latum* or as unspecified *Diphyllobothrium* infections. However, recent data indicate that some of these infections, especially when diagnosed solely on the basis of morphology, have been misidentified. It is thus highly probable that prevalence of other human-infecting *Diphyllobothrium* species is currently underestimated. A molecular diagnostic based on genetic markers such as *cox1* gene has helped identify new cases of diphyllobothriosis in non-endemic regions, which would indicate import of these parasites to new geographical areas. This would be the case in recent *D. latum* re-emergence in the Alpine region of Central Europe (France, Italy and Switzerland) [1]–[4].

*Diphyllobothrium latum* has circumboreal distribution, with most cases reported in northern Europe, Russia (Karelia, Siberia, and Far East), and North America (Canada and Alaska), but has been recently found also in South America (Chile) [1]. Another human-infecting species, *D. nihonkaiense*, seems to dominate the northern Pacific region, whereas *D. pacificum* (Nybelin, 1931) is endemic to the southern Pacific (coast of South America) [1]. Another widely distributed species, *D. dendriticum* (Nitzsch, 1824), has never been considered as an important or frequent parasite of man [5], [6], even though it is rather common in arctic regions [7]. This cestode typically parasitizes arctic and subarctic piscivorous birds (such as gulls) and mammals (such as foxes or bears); human infections have been generally considered accidental [1].

The literature was searched in Rosenberg (1977) [8] and in online databases *Web of Knowledge* and *Pubmed* with the key words “*Diphyllobothrium*” and “*Diphyllobothrium dendriticum*” (July 2013).

## Brief Historical Overview

*Diphyllobothrium dendriticum* was originally described as *Bothriocephalus dendriticus* from *Larus tridactylus* (now *Ricca tridactyla*) by Nitzsch (1824) in northern Germany [9]. The first documented case of human infection with this tapeworm was reported by Cholodkovsky (1916) as *Dibothriocephalus minor* from a man living on the banks of Lake Baikal, Russia [10]. Another species described from man in Russia was *Diphyllobothrium strictum* by Talysin (1932) on Olkhon Island of Lake Baikal and *Diphyllobothrium nenzi* Petrov, 1938 from the lower Pechora River [11], [12]. However, all these species are considered to be synonyms of *D. dendriticum* because they are morphologically indistinguishable [13]–[16].

Thus, most diphyllobothriosis cases caused by *D. dendriticum* were reported from the region of Siberia, especially from the surroundings of Lake Baikal, along with a few from north Canada and Alaska [5], [14]. In contrast, there have been no reliable records of autochthonous human infection in Europe, and all recent reports from Europe most probably represent imported infections (see below) [1], [7].

The actual proportion of diphyllobothriosis caused by *D. dendriticum* is difficult to estimate from the literature because voucher material has rarely been deposited in museum collections for later scrutiny and is seldom suitable for molecular analyses (samples fixed in formalin or AFA, i.e., a mixture of alcohol, formalin, and acetic acid). In addition, several species of *Diphyllobothrium* may occur sympatrically, such as *D. latum* and *D. dendriticum* in Siberia, and *D. alascense* Rausch et Williamson, 1985, *D. dalliae* Rausch, 1956, *D. dendriticum*, *D. latum*, *D. ursi* Rausch, 1954, and recently recognised *D. nihonkaiense* in North America, which further complicates correct diagnosis [1], [17].

## Recent Human Cases

Two clinical cases of *D. dendriticum* infection from Switzerland were confirmed recently by molecular methods [18], [19]. One case was reported from a 59-year-old woman who had visited Norway, Canada, and Alaska one and six years before the infection was detected, respectively. This woman also consumed fish frequently. The other case was a four-year-old boy who had visited tropical Asia and ate smoked or poorly cooked fish there. He had also consumed imported Atlantic salmon (*Salmo salar*) from Norway and perch (*Perca fluviatilis*) from Switzerland.

**Figure 1:**
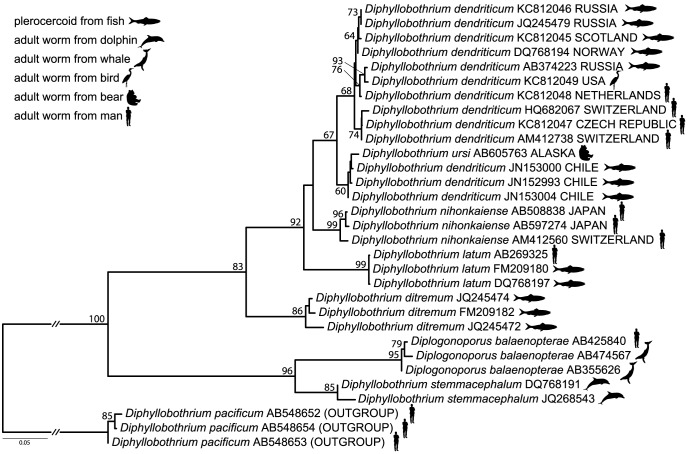
Maximum likelihood (ML) estimate of *Diphyllobothrium dendriticum* phylogenetic relationships based on currently available cox*1* sequences of human-infecting *Diphyllobothrium* species and their close relatives computed in Garli 2.0. Nucleotide data matrix was 1563; codon positions were analyzed separately according to the partition scheme and models (TrN+I) (F81) (TrN+I+G) chosen according to the BIC in PartitionFinder 1.0.1. Nodal support values depict bootstrap support proportions >50 based on 1,000 repetitions estimated in Garli. Note that the *D. pacificum* branch was shortened by a factor of two. Newly obtained sequences are shown in bold type; country of origin is listed for *D. dendriticum* infections.

The third case, falsely assigned as a *D. latum* infection, was reported from the Netherlands. Subsequent molecular phylogenetic analysis proved that the 31-year-old man was infected with *D. dendriticum* (present data, Figure 1). This man had visited Brazil five months prior to the infection was detected and successfully treated, but anamnestic data do not allow to make a safe conclusion on the origin of the infection [20].

**Figure 2:**
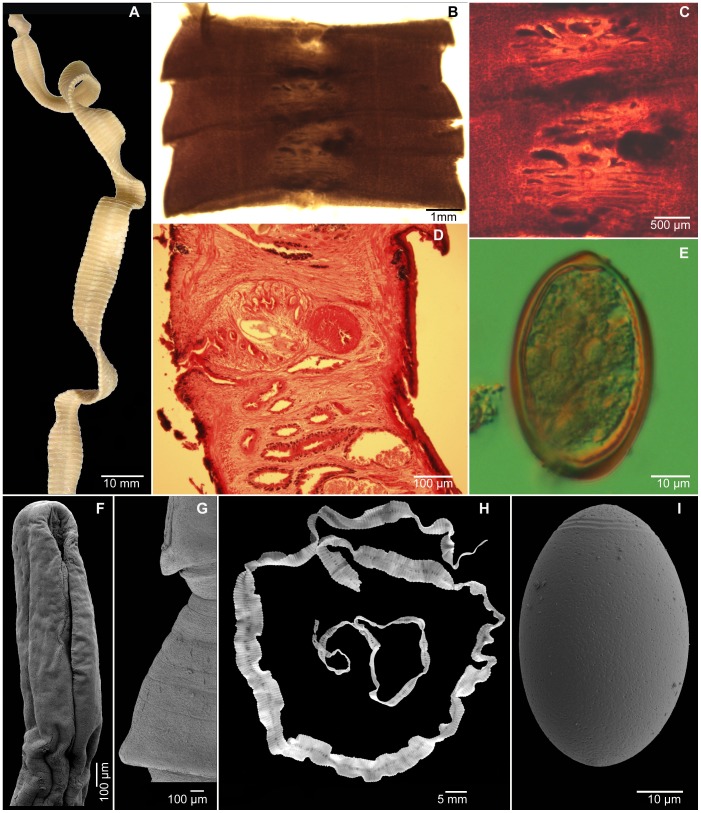
Morphology of *Diphyllobothrium dendriticum*. (A–E, G, H) Human case from the Czech Republic. (A) Whole worm. (B, C) Whole mount of gravid proglottids and their detail. (D) Sagittal section of gravid proglottids. (E, I) Egg in light microscope and scanning electron micrograph (SEM). (F) Scolex (SEM). (G) Lateral extremities of gravid segments (SEM). (H) Whole worm. (F, H) SEM of the specimen from experimentally infected hamster.

The most recent case was recorded in the Czech Republic. A 28-year-old woman that had spent summer 2010 as a seasonal worker in a fish processing plant located in Klawock, southeast Alaska, found a large piece of a tapeworm strobila (∼30 cm) on Jan 5, 2011 (Figure 2). She was treated by a single dose of praziquantel (Cesol), 750 mg, on that day, and the three subsequent stool laboratory checks on January 10–13 returned negative. She acknowledged eating barbecued fish (especially sockeye and pink salmon, *O. nerka* and *O. gorbuscha*) and occasionally other wild salmonids like Arctic cisco (*Coregonus autumnalis*) during her summer job. Molecular phylogenetic analyses based on sequences of the complete cytochrome c oxidase subunit 1 (*cox1*) and partial large subunit nuclear ribosomal DNA (lsrDNA) genes unequivocally determined the cestode as *D. dendriticum* (Figure 1).

## Morphology and Differential Diagnosis

*Diphyllobothrium dendriticum* is a large tapeworm, with body (strobila) length reaching up to 1 m and width up to 1 cm, respectively (Figure 2). The strobila consists of several hundred segments (proglottids), each containing one set of male and female genital organs (Figure 2). Depending on the host and physiological state, the body can vary considerably in size and shape [5]. The head (scolex) is usually spatulate in shape, but its shape varies according to the state of contraction. Neck (proliferation zone between the scolex and strobila) is present in relaxed specimens. Gravid segments, i.e., segments containing fully formed eggs in the uterus, are usually wider (0.82–10.0 mm) than long (0.13–2.1 mm) and have concave lateral margins, with more or less pointed projections formed bilaterally at each segmental junction (Figure 2) [5].

Testes are spherical, numerous, in the medulla (i.e., internal to the inner longitudinal musculature), and confluent between segments and across the anterior margin of the segment. The cirrus-sac (terminal part of the male reproductive organ) is round in dorsoventral view, but oblique in sagittal section (Figure 2). The external seminal vesicle is small, less than half-size of the cirrus-sac, muscular, dorsal to the cirrus-sac, and not visible from the ventral side (Figure 2). The common genital pore (joint opening of the cirrus-sac and vagina) is median, at about anterior third (21–37%) of the length of the segment, situated on an elevation densely covered with papillae (nipples), often elliptical in shape (Figure 2).

The ovary is bilobed, near the posterior margin of the segment, and variable in shape. Vitelline follicles are numerous, small, spherical, situated in the cortex (i.e. external to the inner longitudinal musculature), and confluent between segments and across the anterior margin of the segment. The uterus is tubular, forms six to eight loops reaching up to the cirrus-sac, opening on the ventral side of the segment at its midline, immediately posterior to the genital pore. The eggs are thick-shelled, operculate, 50–70 µm long, and 30–52 µm wide; their surface is almost smooth with a few shallow pits (Figure 2) [14], [18], [19, present study].

Routine diagnostics of diphyllobothriosis is mainly based on the finding of the eggs in stool samples. The size and shape of the operculate eggs, with their minute terminal knobs, are characteristic for the genus, but their species identification is usually impossible because of high intraspecific variation [17], [21], [22]. The species of *Diphyllobothrium* are most readily distinguished by the shape and size of the scolex, neck, and male genital organs (visible in sagittal sections), i.e., morphological features that are not possible to evaluate in most clinical cases (the scolex and neck are rarely found and proglottides are deformed or decomposed as a result of treatment with anthelminthics or inappropriate sample processing).

*Diphyllobothrium dendriticum* differs from the remaining human-infecting species (*D. latum*, *D. nihonkaiense*, and *D. pacificum*) by featuring more concave lateral margins of gravid segments, a more dorsal external seminal vesicle in relation to the cirrus-sac (not visible from the ventral side) and some segments with vitelline follicles confluent at its anterior part [5]. Recently, a molecular test based on multiplex PCR of mitochondrial *cox1* gene has been developed, allowing for a quick differential diagnosis between the common human-infecting *Diphyllobothrium* species [23].

## Molecular Diagnosis and Systematics

Molecular diagnostic methods based on analyses of the *cox1* gene sequences proved capable of reliably distinguishing among the species of human-infecting broad fish tapeworms. The *cox1* gene represents a widely used molecular barcoding marker for species determination of various groups of animals, whose elevated rate of sequence evolution allowed for accumulation of a sufficient number of nucleotide substitutions that are capable of distinguishing one tapeworm species from another. Even a partial sequence of this gene is able to determine the correct species. However, universal primers that amplify the entire *cox1* gene of any *Diphyllobothrium* species exist and should preferably be used, as they provide significantly more data. Species-specific primers that anneal at distinct positions of the *cox1* gene then form the basis of a molecular diagnostic method based on multiplex PCR that allows for rapid differential diagnosis of the human-infecting *Diphyllobothrium* species [23].

Phylogenetic analyses have continuously indicated a close relationship of *D. dendriticum*, *D. latum*, and *D. nihonkaiense*. The last common human parasite, *D. pacificum*, is apparently a more distantly related taxon, most probably forming a basal lineage of the genus *Diphyllobothrium* [3], [23]–[27]. Out of the molecular markers used, *cox1* performs best in both phylogenetic reconstructions and comparative diagnostic analyses. Just recently, the phylogenetic status of *D. ursi*, another human-infecting species described by Rausch (1954) from bear (*Ursus arctos*) in Alaska, was assessed [28], [29]. In these analyses inferred from *cox1* gene sequences, validity of this North American species was supported [29].

A phylogenetic tree based on currently available *cox1* sequences supplemented by new data on several isolates from intermediate and definitive hosts (5 new sequences; see Table 1) suggests a sister-group relationship of *D. dendriticum* and *D. nihonkaiense*, *D. latum* being the sister of the two (Figure 1). However, this branching pattern never gets statistically supported and tends to change according to the method of analysis. The present analysis also questioned the correct determination of *Diphyllobothrium dendriticum* samples from *Oncorhynchus mykiss* from Chile that formed a distinct lineage with *D. ursi* apart from the remaining *D. dendriticum* representatives [30]. Based on the current data, however, it is not possible to test if *D. dendriticum* from Chile was in fact misdiagnosed *D. ursi* or if *D. ursi* represents a large form of *D. dendriticum* from bears (Figure 1).

**Table 1:** Sequences of *Diphyllobothrium dendriticum* used in phylogenetic analysis (Figure 1).

Access. No.	Stage	Host	Locality (possible origin in parentheses)	Authority
KC812046	plerocercoid	*Coregonus autumnalis*	Lake Baikal, Russia	new sequence
JQ245479	plerocercoid	*Coregonus autumnalis*	Lake Baikal, Russia	Suleymanov et al. (unpublished article)
KC812045	plerocercoid	*Coregonus lavaretus*	Loch Lomond, Scotland, UK	new sequence
DQ768194	plerocercoid	*Salvelinus alpinus*	Fjellfrosvatn Lake, Norway	Yera et al. 2008 [3]
AB374223	plerocercoid	*Salvelinus leucomaenis*	Lake Azabachye, Kamchatka, Russia	Arizono et al. 2009 [53]
KC812049	adult	*Larus hyperboreus*	Kansas, USA	new sequence
KC812048	adult	*Homo sapiens*	Netherlands (Brazil)	new sequence**
HQ682067	adult	*Homo sapiens*	Switzerland	de Marval et al. 2013 [19]
KC812047	adult	*Homo sapiens*	Czech Republic (Alaska)	new sequence
AM412738	adult	*Homo sapiens*	Bern, Switzerland	Wicht et al. 2008 [18]
AB605763*	adult	*Ursus arctos middendorffi*	Kodiak Island, Alaska, USA	Yamasaki et al. 2012 [29]
JN152993*	plerocercoid	*Oncorhynchus mykiss*	Tarahuin Lake, Chile	Rozas et al. 2012 [30]
JN153000*	plerocercoid	*Oncorhynchus mykiss*	Tarahuin Lake, Chile	Rozas et al. 2012 [30]
JN153004*	plerocercoid	*Oncorhynchus mykiss*	Natri Lake, Chile	Rozas et al. 2012 [30]

sequences of *Diphyllobothrium ursi*;

see van Doorn et al. 2005 [20].

The origin of *D. dendriticum* in South America is not known. It has been probably imported by migratory birds such as *Sterna hirundo*, *S. paradisea*, and *Larus pipixcan* on their visits to South America [31]. Completing of the life-cycle was most likely possible due to the introduction of the second intermediate host—rainbow trout *O. mykiss—*at the beginning of the 20th century. However, native fish, such as *Galaxias maculatus*, *G. platei*, *Diplomystes composensis*, *Percichthys trucha* and several others are also infected with *Diphyllobothrium* plerocercoids in Chile [32].

## Life Cycle and Epidemiology

The life cycle of *D. dendriticum* is similar to life cycles of other *Diphyllobothrium* species, all of which include three hosts [1]. Planktonic copepods serve as the first intermediate hosts in which the larval stage, called procercoid, develops. The second larval stage or metacestode, called plerocercoid, develops in freshwater and anadromous fishes, especially salmonids [5]. However, *D. dendriticum* plerocercoids were found in more than 50 species of 12 families of freshwater fish (Abyssocottidae, Atherinopsidae, Balitoridae, Comephoridae, Cottidae, Cottocomephoridae, Gadidae, Galaxiidae, Gasterosteidae, Osmeridae, Percichthyidae, and Salmonidae) [33]. Despite this extraordinarily wide spectrum of fish hosts, *D. dendriticum* has never been reported from naturally or experimentally infected perch (*Perca fluviatilis*) or pike (*Esox lucius*), which are the principal second intermediate hosts of *D. latum* in the Palaearctic region [5], [34].

Plerocercoids of *D. dendriticum* are usually encysted within the visceral organs or body cavity, whereas records of free larvae in the muscles of Pacific salmons and trouts such as *Oncorhynchus nerca* and *O. clarki* should be considered doubtful unless verified using molecular markers [5], [35]. Humans can become infected either by consuming raw or undercooked visceral organs, e.g., liver and ovaries, or flesh of fish that were gutted, but where plerocercoids remained on the ventral abdominal flap attached to the fillet or migrated to the musculature [36], [37].

Adults of *D. dendriticum* have been found in birds of nine families (Accipitridae, Alcidae, Corvidae, Gaviidae, Laridae, Pandionidae, Pelecanidae, Podicipedidae, and Sternidae), with the majority of the records coming from gulls (Laridae) [38], [39], [33]. The prevalence of infection in these principal definitive hosts is usually low (1–25%) [40]–[43], except for gulls in the endemic area of Baikal, where up to 76% *Larus argentatus* were infected [13], [39], [44]. Common definitive hosts are also mammals, such as the arctic fox (*Alopex lagopus*) with prevalence of 4–15% reported from Greenland and Iceland [45]–[47].

The prepatent period of *D. dendriticum* is short, less than two weeks in man, with maximum egg shedding in late summer and fall [6], [7], [36]. The parasite longevity in the definitive host is assumed to last only four to six months [7], but Wicht et al. (2008) reported a patient infected with *D. dendriticum* with two years of chronically relapsing diarrhoea [18].

## Geographical Distribution and Endemic Areas

**Figure 3:**
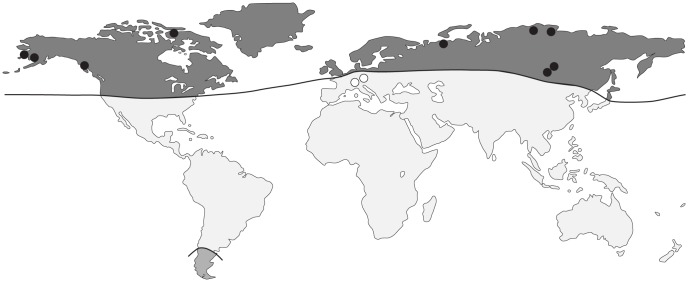
Geographical distribution and human cases of *Diphyllobothrium dendriticum*. Black dots represent autochthonous human cases; white dots represent imported human cases. Black line delimits the area of *D. dendriticum* distribution (grey colour).

The original distribution of *D. dendriticum* is circumboreal, but the parasite was allegedly found also in trout introduced to Argentina and Chile (see Molecular Diagnosis and Systematics) (Figure 3) [30], [31], [48]. The geographical distribution of *D. dendriticum* and *D. latum* overlaps; however, *D. dendriticum* tends to predominate in arctic regions, where it infects salmonids and coregonids, whereas *D. latum* infections are characteristic for more subarctic and temperate areas (Figure 3) [7].

In the Lake Baikal region, the prevalence of cases with *D. dendriticum* decreased markedly from almost 30% in 1929 to less than 0.01% in 2005–2007 [13], [49], however, the prevalence in other regions of Russia (Buryatia, Krasnoyarsk district, Taymyr Peninsula, and Yakutia) remains rather high (up to 14%) [49]–[51] (2012 email from I. Kutyrev, Ulan-Ude to the senior author; unreferenced).

Another endemic area of *D. dendriticum* is arctic North America, but the number of cases is much lower compared to Siberia [5], [17]. At least ten human records have been confirmed from Alaska, Nunavut and British Columbia, mostly from native Inuit populations, but the real numbers are unknown [5, present study].

## Medical Importance and Control

Diphyllobothriosis is not a life-threatening disease and most human cases are mild or even asymptomatic [1]. Since the causative agents of numerous clinical cases have not been reliably identified, it is not possible to distinguish differences in the pathogenicity of individual species, including *D. dendriticum*. However, we assume that *D. dendriticum* does not represent a serious human pathogen that would cause disease with severe clinical signs. Nevertheless, some infections can result in chronically relapsing diarrhoea and thus require the attention of medical doctors and adequate treatment [18].

Rolf Vik infected himself with *D. dendriticum* while in the United States working on *Oncorhynchus clarkii* in order to take the adult tapeworms back to Norway to make comparisons with Norwegian species. He did not observe any health problems [5].

Treatment of diphyllobothriosis is effective, with praziquantel being the drug of choice. Prophylaxis is also straightforward—the key measure is to avoid consumption of raw or undercooked fish. However, consumption of raw or lightly pickled fish is considered a traditional delicacy by numerous nationalities and this habit has gained ground quickly around the world.

Prevention of water contamination through waste water purification in sewage plants represents another way of controlling the disease. However, its impact is limited in the case of *D. dendriticum* because a variety of definitive hosts might serve as reservoirs of the disease in a given area. The elevated mobility of these reservoir hosts (especially piscivorous birds) enables the parasite to disseminate eggs over large areas, thus representing a serious obstacle in the control of diphyllobothriosis caused by *D. dendriticum*.

Climate change will result in faunal shift (global warming) and will influence parasites, especially those that undergo temperature-dependent development [52]. This also concerns broad fish tapeworms (*Diphyllobothrium* spp.), including *D. dendriticum*, the life cycle of which is realized in a freshwater environment (first intermediate hosts are planktonic copepods and second intermediate hosts are teleost fishes). Climate change and global warming certainly represent new challenges to assess their impact on ecosystems, including aquatic ones, with corresponding impact on parasite distribution [52].

## Conclusions

Parasitic infections caused by tapeworms (Cestoda) do not generally represent a serious public health concern in developed countries, with relatively very few exceptions such as echinococcosis, sparganosis, and cysticercosis,. Nevertheless, several food-borne diseases and/or zoonoses have emerged during the last decades as a result of global trade (transport of fresh fish “on the ice”), increased mobility of people, and their changing eating habits, i.e., increased popularity of raw or undercooked food. The tapeworm *Diphyllobothrium dendriticum* represents an example of a previously neglected, probably underdiagnosed parasite of man with potential to spread globally.

Recent cases of diphyllobothriosis caused by *D. dendriticum* in Europe (Netherlands, Switzerland and Czech Republic), where the parasite has not been reported previously, represent evidence that causative agents of zoonoses can be imported throughout the world. It is thus necessary to pay attention also to previously rare or non-native parasites. Molecular tools should be used for specific and reliable diagnostics, which may considerably help improve our knowledge of the distribution and epidemiology of these human parasites.

**Key Learning Points**

- On the basis of revision of more than 900 available references and a description and revision of recent European human cases using morphological data, we updated the current knowledge of the life cycle, geographic distribution, and epidemiological status of this emerging causal agent of zoonotic disease of man.
- The molecular (*cox1*) data supplemented by five newly characterized *D. dendriticum* sequences and molecular diagnostics of this emerging disease were added and discussed.
- The tapeworm *Diphyllobothrium dendriticum* represents an example of a previously neglected, probably underdiagnosed parasite of man with a potential to spread globally.

**Five Key Papers in the Field**

1. Scholz T, Garcia HH, Kuchta R, Wicht B (2009) Update on the human broad tapeworm (genus *Diphyllobothrium*), including clinical relevance. Clin Microbiol Rev 22: 146–160.
2. de Marval F, Gottstein B, Weber M, Wicht B (2013) Imported diphyllobothriasis in Switzerland: molecular methods to define a clinical case of *Diphyllobothrium* infection as *Diphyllobothrium dendriticum*, August 2010. Euro Surveill 18: 31–36.
3. Wicht B, Ruggeri-Bernardi N, Yanagida T, Nakao M, Peduzzi R, et al. (2010) Inter- and intra-specific characterization of tapeworms of the genus *Diphyllobothrium* (Cestoda: Diphyllobothriidea) from Switzerland, using nuclear and mitochondrial DNA targets. Parasitol Int 59: 35–39.
4. Dupouy-Camet J, Peduzzi R (2004) Current situation of human diphyllobothriasis in Europe. Eurosurveillance 9: 31–34.
5. Curtis MA, Bylund G (1991) Diphyllobothriasis: fish tapeworm disease in the circumpolar north. Arctic Med Res 50: 18–25.
